# Epithelial-Mesenchymal Transition in Cells Expanded *In Vitro* from Lineage-Traced Adult Human Pancreatic Beta Cells

**DOI:** 10.1371/journal.pone.0006417

**Published:** 2009-07-29

**Authors:** Holger A. Russ, Philippe Ravassard, Julie Kerr-Conte, Francois Pattou, Shimon Efrat

**Affiliations:** 1 Department of Human Molecular Genetics and Biochemistry, Sackler School of Medicine, Tel Aviv University, Ramat Aviv, Tel Aviv, Israel; 2 Biothechnology and Biotherapy Laboratory, Research Center of the Brain and Spinal Cord Institute, CNRS UMR 7225, INSERM UMRS 975, and University Pierre and Marie Curie, Hôpital Pitié Salpêtrière, Paris, France; 3 U859 Diabetes Cell Therapy, University Lille-Nord de France, Lille, France; University of Bremen, Germany

## Abstract

**Background:**

*In-vitro* expansion of functional beta cells from adult human islets is an attractive approach for generating an abundant source of cells for beta-cell replacement therapy of diabetes. Using genetic cell-lineage tracing we have recently shown that beta cells cultured from adult human islets undergo rapid dedifferentiation and proliferate for up to 16 population doublings. These cells have raised interest as potential candidates for redifferentiation into functional insulin-producing cells. Previous work has associated dedifferentiation of cultured epithelial cells with epithelial-mesenchymal transition (EMT), and suggested that EMT generates cells with stem cell properties. Here we investigated the occurrence of EMT in these cultures and assessed their stem cell potential.

**Methodology/Principal Findings:**

Using cell-lineage tracing we provide direct evidence for occurrence of EMT in cells originating from beta cells in cultures of adult human islet cells. These cells express multiple mesenchymal markers, as well as markers associated with mesenchymal stem cells (MSC). However, we do not find evidence for the ability of such cells, nor of cells in these cultures derived from a non-beta-cell origin, to significantly differentiate into mesodermal cell types.

**Conclusions/Significance:**

These findings constitute the first demonstration based on genetic lineage-tracing of EMT in cultured adult primary human cells, and show that EMT does not induce multipotency in cells derived from human beta cells.

## Introduction

*In-vitro* expansion of functional beta cells from the limited number of donated adult human pancreata is an attractive approach for generating an abundant source of cells for beta-cell replacement therapy of diabetes. Despite evidence supporting the replicative capacity of both rodent and human beta cells *in vivo* [1]–[4], attempts at expanding human islet cells *in vitro* resulted in limited replication and loss of beta-cell phenotype [5]–[7]. To monitor the fate of cultured human beta cells we developed a lineage-tracing approach based on a Cre-loxP-mediated DNA recombination system delivered by lentivirus vectors [8]. Using this system >50% of the insulin-positive cells present in isolated human islets could be specifically labeled with enhanced green fluorescent protein (eGFP). The labeled beta cells were shown to undergo rapid dedifferentiation and proliferate readily for up to 16 population doublings. The percent of eGFP^+^ cells remained stable during the entire expansion period, suggesting a comparable replication rate of eGFP^+^ and eGFP^−^ cells [8]. In contrast to human beta cells, labeling of mouse beta cells by transgenic methods [9]–[12], as well as by our lentivirus method [8], showed that mouse beta cells did not significantly proliferate under these culture conditions.

Dedifferentiation of epithelial cells has been associated with epithelial-mesenchymal transition (EMT) in cultured thyroid cells [13]. EMT plays a key role in morphogenic changes during embryonic development and in tumor metastasis (see ref. [14] for a recent review), however its role in cell dedifferentiation remains unclear. Furthermore, previous work has suggested that EMT generates cells with stem cell properties [15]. Gershengorn et al. postulated that beta cells in cultures of adult human islets underwent EMT upon entrance into the cell cycle [16], however, in the absence of cell lineage-tracing there was no direct evidence for the origin of mesenchymal cells from beta cells in these cultures. Subsequent work from this group abandoned the EMT hypothesis and suggested instead that cells expanded in human islet cultures, termed human Islet Progenitor Cells (hIPC), were derived from rare mesenchymal stem cells (MSC) normally present in the islets. hIPCs expanded *in vitro* were shown to express MSC markers, and a fraction of them differentiated *in vitro* into mesodermal cell types, such as adipocytes and osteocytes [17]. The presence of MSC in some human islet preparations was supported by another group [18]. However, the presence of MSC in islets *in vivo* has not been confirmed, and their occurrence in preparations of isolated islets may result from contaminating pancreatic exocrine and duct tissue [19].

Our lineage tracing approach allows direct evaluation as to the occurrence of EMT in cultured human beta cells. Here we report that cells originating from beta cells undergo EMT in cultures of adult human islet cells and express multiple mesenchymal markers, as well as markers associated with MSC. However, we do not find evidence for the ability of such cells, nor of other cells in these cultures expressing MSC markers, which are derived from a non-beta-cell origin, to significantly differentiate into mesodermal cell types. These findings constitute the first demonstration based on genetic lineage-tracing of EMT in cultured adult primary human cells, and show that EMT does not induce multipotency in cells derived from human beta cells.

## Materials and Methods

### Ethics statement

This study was conducted according to the principles expressed in the Declaration of Helsinki. The Institutional Review Boards of the medical centers, which provided human islets (members of the Islet Cell Resource Consortium) and bone marrow, each provided approval for the collection of samples and subsequent analysis. All donors provided written informed consent for the collection of all samples and subsequent analysis.

### Vector construction and production

The reporter lentiviral vector was modified from pTrip CMV-loxP-DsRed2-loxP-eGFP DeltaU3 [8] as follows. The DsRed2 sequence was removed and replaced by the reading frame A Gateway cassette (Gateway Conversion Kit, Invitrogen), upstream of a stop linker composed of 6 stop codons placed as pairs in the 3 reading frames, resulting in pTrip–loxP-Gateway-STOP-loxP-eGFP DeltaU3 destination vector. The Neomycin resistance gene was amplified by PCR from pcDNA3 vector using the forward primer 5′CACCATGATTGAACAAGATGGA3′ and reverse primer 5′GAAGAACTCGTCAAGAAGGCGA3′, and the resulting PCR product was cloned into the pENTR/D/TOPO plasmid (Invitrogen) to generate a Neomycin entry clone. Both entry clone and destination vector were used for *in-vitro* recombination mediated by LR clonase II (Invitrogen), resulting in a new reporter lentiviral vector, pTrip–loxP-NEO-STOP-loxP-eGFP DeltaU3. A tamoxifen-inducible form of Cre (Cre-ERT2) was cloned downstream of the 405-bp fragment of the rat insulin promoter (RIP 405). Briefly, LR clonase II recombination was performed using pTrip RIP405 rfa-Gateway DeltaU3 destination vector [8] and pENTR/D/TOPO–Cre-ERT2 entry clone. The Cre-ERT2 fragment was amplified by PCR from a plasmid kindly provided by Guilan Vodjdani using the forward primer 5′CACCGGTACCCTCGAGATCGAT3′ and reverse primer 5′TCAAGCTGTGGCAGGGAAACC3′, and the resulting PCR product was cloned into the pENTR/D/TOPO plasmid to generate the Cre-ERT2 entry clone. Virus particles were produced in 293T cells following vector cotransfection with the pCMVdR8.91 and pMD2.G plasmids. The culture medium was harvested 36–48 h later.

### Cell culture and infection

Human islets were received in our laboratory 3.1±1.4 days following isolation. Islet purity was determined by staining with dithizone. Islets from individual donors were dissociated into single cells and cultured as described [20]. Following 1 day in culture cells were washed with PBS and infected with a 1∶1 mixture of the 2 viruses at MOI 3∶1 in CMRL containing 8 µg/ml polybrene overnight. The medium was replaced in the morning, and 4-hydroxy-tamoxifen (Sigma-Aldrich, St.Louis, MO) was added to a final concentration of 1 µM in the evening. Following overnight incubation the medium was changed to regular growth medium. Human bone marrow cells were obtained from adult donors at Lanadio Hospital under approved protocols. Human bone marrow derived MSC (BM-MSC) were isolated and cultured as described [21].

### Cell sorting and flow cytometry

Labeled cells were sorted using a FACS Aria cell sorter (Becton Dickinson, San Jose, CA) with a fluorescein isothiocyanate (FITC) filter (530/30 nm) for eGFP. Dead cells stained with 7-amino actinomycin D (7-AAD, Invitrogen) were excluded using a PerCP-Cy5.5 filter (695/40 nm).

### RNA analysis

Total RNA was isolated using Trizol (Sigma-Aldrich, St.Louis, MO) and treated with DNA-free (Ambion, Redwood, TX) to remove genomic DNA. cDNA was prepared using High Capacity cDNA RT Kit (Applied Biosystems, Foster City,CA). cDNA quantitation (qRT-PCR) was performed using the following Assay-on-Demand kits (Applied Biosystems, Foster City, CA): insulin, Hs 00355773 m1; E-Cadherin, Hs 01013953 m1; smooth muscle actin, Hs 00909449 m1; CD73, Hs 00159686 m1; CD90, Hs 00174816 m1; CD105, Hs 00164438 m1; N-Cadherin, Hs 00169953 m1; vimentin, Hs 00185584 m1; RPLP0, Hs 99999902 m1. All reactions were done in triplicates. The results were normalized to human large ribosomal protein P0 cDNA (RPLP0).

### Immunofluorescence

Cells grown on sterilized coverslips were washed with PBS and fixed with 4% paraformaldehyde (PFA) for 10 min at room temperature. Slides were permeabilized for 10 min with 0.25% NP40. Cells were blocked for 40 min with 5% fetal goat serum, 1% bovine serum albumin and 0.2% saponin and incubated overnight at 4°C with the primary antibodies diluted in blocking solution. Cells were then washed and incubated for 40 min with the secondary antibodies. Images were taken using a Zeiss LTM 200 Apotome or a Leica SP-5 confocal microscope. Expression of eGFP was detected using mouse anti-GFP (Chemicon, 1∶500) or rabbit anti-GFP (Invitrogen, 1∶1000). Other primary antibodies used: mouse anti-human CD90 (BD Pharmingen, 1∶100), mouse anti-human CD105 (BD Pharmingen, 1∶100), mouse anti-smooth muscle actin (Progen, 1∶250), rabbit anti-N-Cadherin (Abcam, 1∶200), mouse anti-vimentin (Calbiochem, 1∶50) and goat anti-fatty acid binding protein 4 (FABP4) (R&D Systems, 1∶100). Secondary antibodies used: Alexa 488 or Alexa 546 anti-mouse IgG, anti-rabbit IgG, or anti-goat IgG (all Invitrogen, 1∶500). DNA was stained with DAPI (Sigma-Aldrich, St.Louis, MO).

### Induction of MSC differentiation into adipocytes and osteocytes

The differentiation experiments were preformed with 3 different kits (Lonza, Walkersville, MD; StemCell Technologies, Vancouver; R&D Systems, Minneapolis, MN). Briefly, BM-MSC and expanded islet cells were seeded on glass slides at densities indicated by the manufacturers. Thereafter cultures were treated according to the manufactures protocols. To identify lipid vacuoles cells were fixed for 20 min in 4% PFA and stained for 30 min in 0.3% Oil Red O (Sigma-Aldrich, St.Louis, MO). Calcium deposition was visualized by fixing cells for 20 min in 4% PFA followed by staining with 0.5% Alizarin Red (Sigma-Aldrich, St.Louis, MO) for 5 min. Following staining with dyes slides were thoroughly washed with water, and nuclei were counterstained using haematoxylin (Pioneer, Essex).

### Statistical analysis

Significance was determined using a two tailed t-test or χ^2^-test. To approach a normal distribution of the quantitative RT-PCR data, a logarithmic transformation was preformed.

## Results

**Figure 1:**
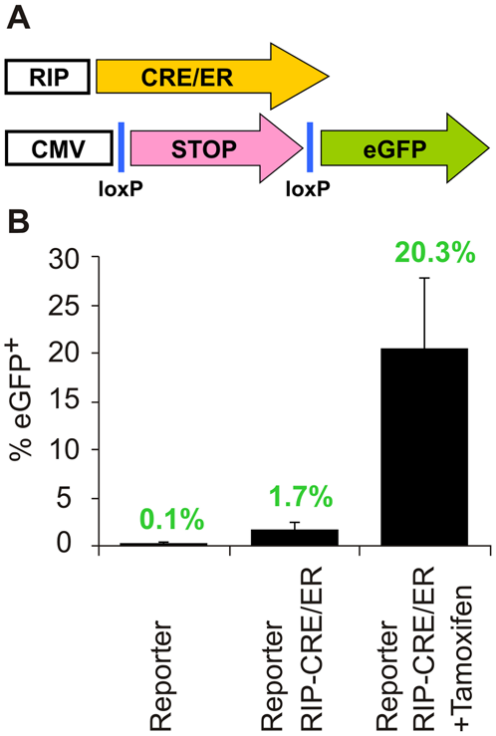
Inducible labeling of human islet cells. A, Schematic representation of the two lentivirus vectors. B, Labeling efficiency and leakiness. Adult human islet cells from 5 donors were infected one day after plating with the reporter virus alone, or with both viruses, and cultured overnight in the absence or presence of tamoxifen. Five days later 10^4^ cells from each donor were analyzed by flow cytometry for eGFP expression. Data are mean±SD (n = 5).

To allow improved labeling of human beta cells for lineage tracing we generated a RIP-Cre/ER lentivirus, in which expression of Cre recombinase is inducible by tamoxifen. In addition, the reporter lentivirus vector was modified to the structure CMVp-loxP-NEO-STOP-loxP-eGFP (Fig. 1). This vector lacks the DsRed2 gene that was present in the previous version, following our finding that DsRed2 expression interfered with sorting of eGFP^+^ cells by FACS. Beta cells infected by both constructs are expected to express Cre protein, however Cre will translocate into the nucleus only upon addition of tamoxifen, leading to removal of the floxed DNA fragment in the reporter construct and activation of eGFP expression. This system provides a labeling efficiency comparable to that of the original labeling system [8], averaging 20.3% of the total islet cells (Fig. 1). (It should be noted that the term “islet cells” refers to the isolated islet preparations, which contain about 20% contaminating pancreatic non-islet cells). Its leakiness in the absence of tamoxifen is relatively low, averaging 1.7% of the total islet cells (Fig. 1). It should be noted that this limited leakiness nevertheless represents specific labeling of beta cells, and does not constitute a drawback in the context of this study. Given the islet purity (81.6±9.4%, based on dithizone staining), and the average of 55% insulin-positive cells among human islet cells [22], <44.9% of the cells could be expected to be insulin-positive at the time of islet isolation. Thus, labeling of 20.3% of the total islet cells represents an efficiency of beta-cell labeling of at least 45.2%, which is comparable to the 57.5±8.9% reported for the original labeling system [8]. Given that dithizone staining is inaccurate, the actual labeling efficiency could deviate somewhat from the one calculated based on dithizone staining.

**Figure 2:**
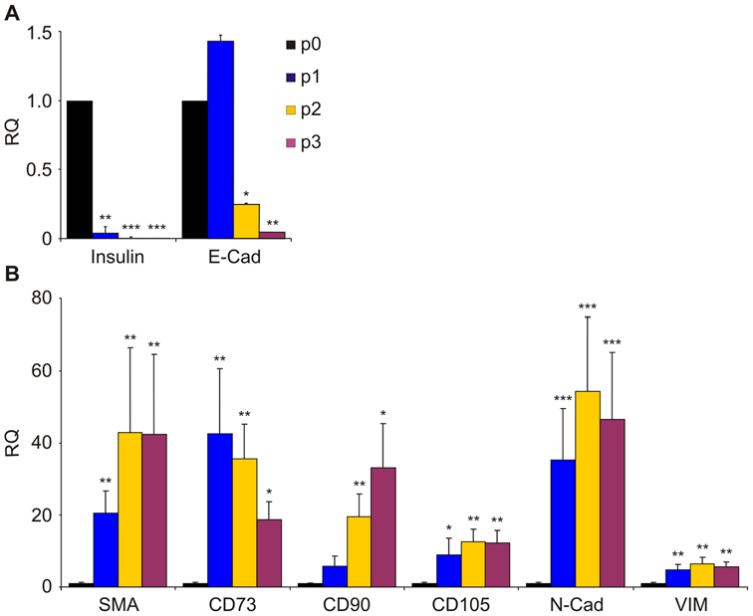
Changes in expression of epithelial and mesenchymal genes in human islet cells during the first 3 weeks in culture. RNA was extracted from cells at the indicated passage number (each passage is equivalent to one week) and analyzed by qRT-PCR. A, analysis of epithelial genes. B, analysis of mesenchymal genes. Data represent relative quantification (RQ) (compared to passage 0) and are mean±SE (n = 6 donors). *p<0.05; ** p<0.005; ***p<0.0005 (compared to passage 0).

Human islets isolated from both male and female donors (age 44.9±12.8; BMI 27.6±5.8) were dissociated into single cells and cultured as described [8]. One day after infection with the two lentiviruses constituting the labeling system cultures were treated with tamoxifen overnight to label the beta cells. They were then expanded and split 1∶2 once a week. Analysis of gene expression in the cultured cells revealed a rapid loss of beta-cell and epithelial phenotypes, as manifested by a large decrease in the levels of insulin and E-cadherin mRNAs (Fig. 2A). In parallel, a pronounced increase in levels of transcripts encoding mesenchymal and MSC markers was detected. These included the mesenchymal markers smooth-muscle actin (SMA), N-cadherin, and vimentin, and the MSC markers CD73, CD90, and CD105 (Fig. 2B). These results were reproducible in cells derived from 6 donors.

**Figure 3:**
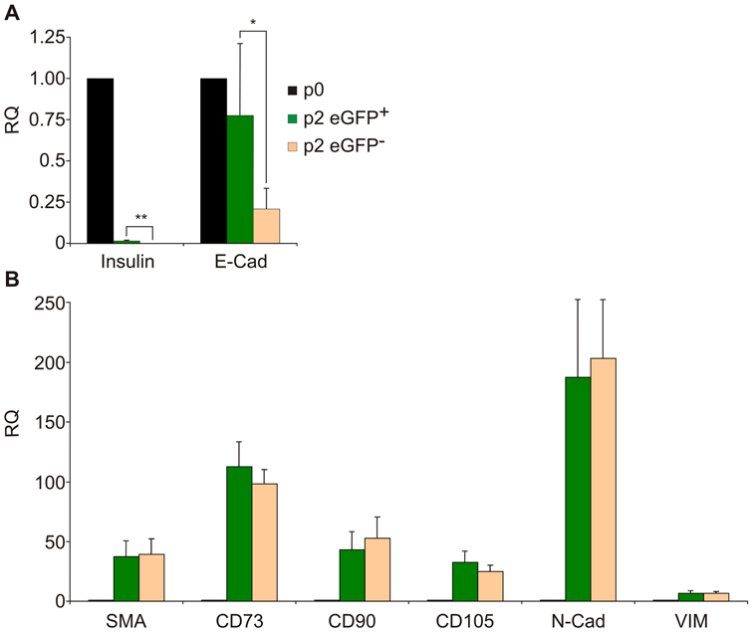
Changes in expression of epithelial and mesenchymal genes in sorted eGFP^+^ and eGFP^−^ cells following 2 weeks in culture. RNA was extracted from cells at the indicated passage number and analyzed by qRT-PCR. A, analysis of epithelial genes. B, analysis of mesenchymal genes. Data represent relative quantification (RQ) (compared to passage 0) and are mean±SE (n = 3 donors). *p<0.05; ** p<0.005.

To evaluate the changes in cells derived from beta cells, eGFP^+^ cells were fractionated by FACS and analyzed for gene expression. This fractionation resulted in >4X enrichment of eGFP^+^ cells, to >82% of the sorted cell population. In the eGFP^−^ population generated by cell sorting the percent of residual eGFP^+^ cells was <7.9%. In addition, this population also contained unlabeled cells derived from beta cells. Thus, while the 2 populations were enriched, they were not pure. Nevertheless, mRNA analyses revealed significant differences in gene expression between the 2 populations. At passage 2 insulin mRNA levels in the sorted eGFP^+^ cells were 84-fold lower, compared with the islet population at passage 0, however they were 36.2±8.9-fold higher, compared with those in eGFP^−^ cells (n = 3 donors, p = 0.002) (Fig. 3A). Similarly, E-cadherin mRNA levels in the sorted eGFP^+^ cells at passage 2 were 5-fold lower, compared with the islet population at passage 0, and 4.5±0.8-fold higher, compared with those in eGFP^−^ cells (Fig. 3A, p = 0.005). In contrast, the mesenchymal and MSC markers analyzed were greatly elevated in both eGFP^+^ and eGFP^−^ cells, and no significant differences were noted between the 2 populations (Fig. 3B). Similar differences were found in cells from 3 donors sorted at passage 3 (data not shown).

**Figure 4:**
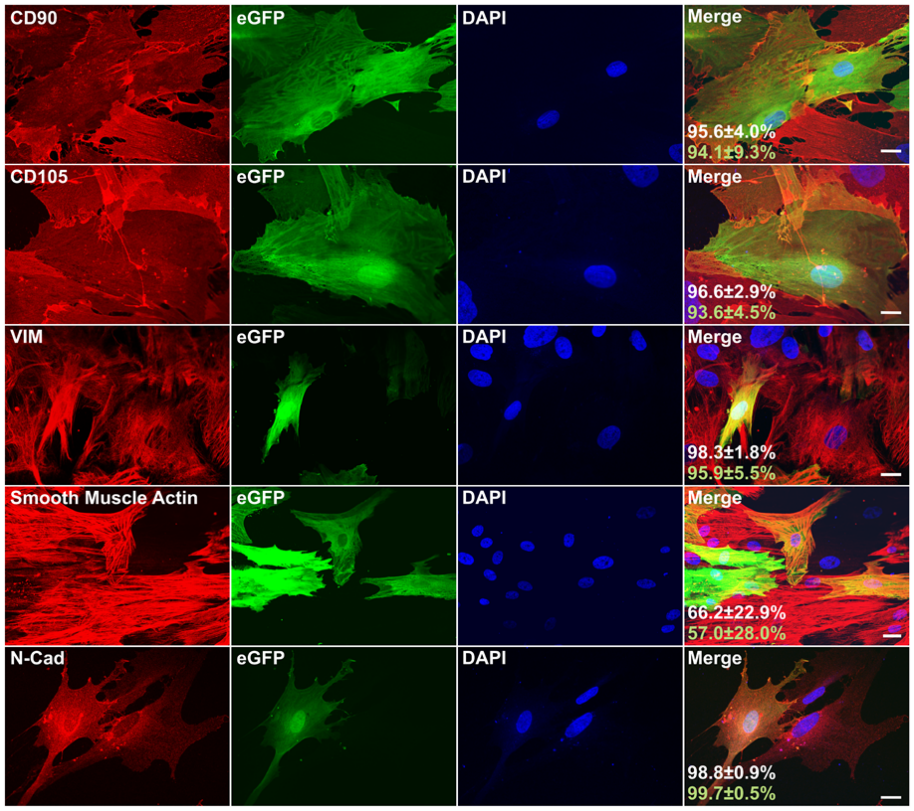
Expression of mesenchymal genes in eGFP^+^ cells. Cells at passage 4 were stained with antibodies to eGFP and the indicated mesenchymal marker. Nuclei were stained blue with DAPI. Bar = 20 µm. The percentages indicate the fraction of cells positive for each mesenchymal marker among eGFP^+^ cells (green digits) and among all the cells (white digits). Data are mean±SD of >400 cells scored from each donor (n = 3 donors).

These analyses suggested that expression of the mesenchymal and MSC markers was greatly elevated in cells derived from beta cells. To directly verify this possibility, labeled cells at passage 4 were stained with antibodies to eGFP and 5 mesenchymal and MSC markers (Fig. 4). Scoring of >400 cells stained for each antigen in cells derived from each of 3 donors revealed that 94.1±9.3% and 93.6±4.5% of eGFP^+^ cells were positive for CD90 and CD105, respectively. Similarly, 99.7±0.5% and 95.9±5.5% of eGFP^+^ cells were positive for N-cadherin and vimentin, respectively. In contrast, in freshly isolated islets, and in islet cells during the first 2 days of culture, only very rare C-peptide^+^ cells were positive for these markers (data not shown). The mesenchymal and MSC markers were also highly abundant in eGFP^−^ cells (Fig. 4), confirming the lack of significant differences in transcript levels for these markers between sorted eGFP^+^ and eGFP^−^ cells (Fig. 3B). Taken together, these findings directly demonstrate the activation of mesenchymal and MSC markers in cells derived from beta cells, thus supporting the occurrence of EMT in these cells.

**Figure 5:**
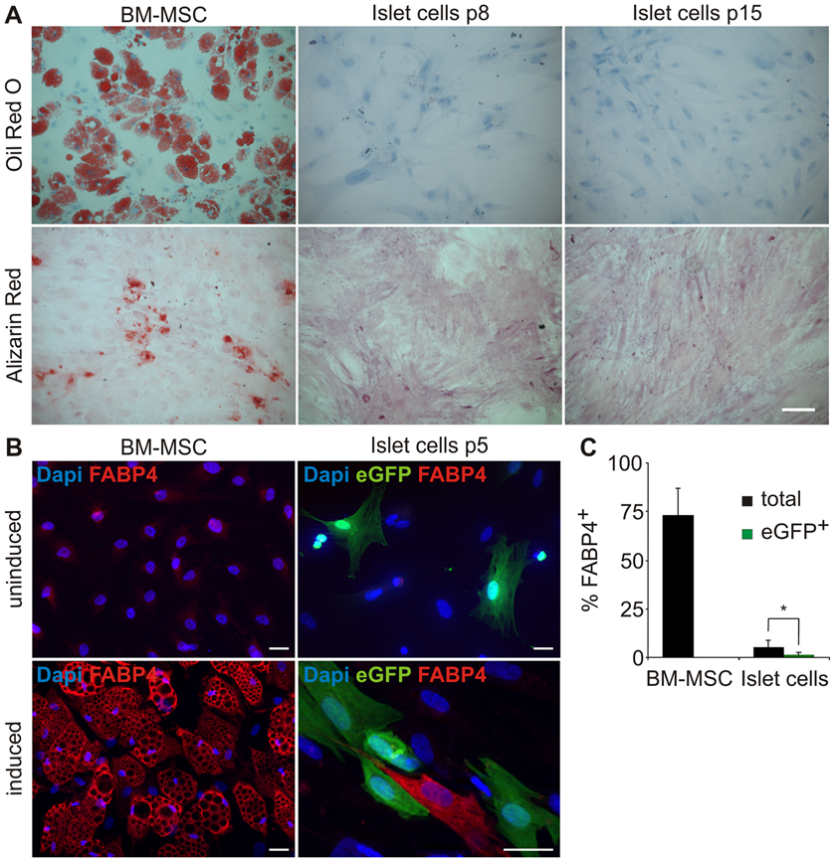
Differentiation of cells expanded from human islet cells into adipocytes and osteocytes. A, Islet cells at the indicated passage number were incubated in Lonza induction medium and stained with Oil Red O for adipocytes and Alizarin Red for osteocytes. Human BM-MSC served as positive control. Bar = 100 µm. B, Islet cells at passage 5 and BM-MSC were incubated in Lonza adipogenesis induction medium and stained with antibodies to eGFP and FABP4. Nuclei were stained blue with DAPI. Bar = 30 µm. The eGFP^+^ cells shown do not stain for FABP4. The single FABP4^+^ cell shown is not eGFP^+^. C, Quantitation of the staining in B, based on counting >500 cells in cultures derived from each donor. Data represent percent of FABP4^+^ among eGFP^+^ cells (green bars) and among all the cells (black bars) and are mean±SD (n = 6 donors for islet cells and 2 donors for BM-MSC). p = 4.18E-14.

To investigate whether the expanded islet cells, which acquired MSC markers, also developed multipotency, they were treated with inducers of adipocyte and osteocyte differentiation. As seen in Fig. 5A, these inducers resulted in efficient differentiation of adult human BM-MSC into adipocytes and osteocytes, as detected by staining with Oil Red O and Alizarin Red, respectively. In contrast, only very rare cells stained with these dyes were detected in cells expanded from adult human islets. To specifically assay the capacity to differentiate into adipocytes in cells derived from beta cells, eGFP^+^ cells were stained with antibodies for the adipocyte marker fatty acid binding protein 4 (FABP4). As seen in Fig. 5B and 5C, 73.1±13.8% of BM-MSC were stained for this marker following induction of differentiation into adipocytes. In contrast, only 5.5±2.9% of all cells expanded from adult human islet cells and induced to differentiate into adipocytes at passages 3–8 were positive for FABP4 (based on counting >500 cells each in cells derived from 6 donors). Among all cells positive for FABP4, only 1.4% were eGFP^+^ cells (representing <0.08% of the total population). When scoring specifically eGFP^+^ cells (a total of 2378 cells counted from 5 donors), 1.9±1.6% were found to be positive for FABP4. Similar results were obtained with 2 additional kits of reagents for induction of MSC differentiation into adipocytes (data not shown). These results demonstrate that cells derived from beta cells, which undergo EMT and acquire MSC markers, are not *bona fide* MSC, as judged by their inability to efficiently differentiate into mesodermal cell types. In addition, the adipocyte and osteocyte differentiation capacity of eGFP^−^ cells in these cultures, the majority of which also express MSC markers and are derived from unknown non-beta-cell sources in the islet preparation, is also quite limited, compared with that of BM-MSC.

## Discussion

These results provide direct evidence for occurrence of EMT in adult human beta cells propagated in culture. This possibility was first suggested by Gershengorn et al. [16], however this group did not present unequivocal data in its support, and eventually backed up from it [17]. Given that nearly all the cells in cultures of human islet cells express mesenchymal markers, and on average about 40% of these cells are derived from beta cells [8], our data suggest that about 40% of the cells expressing mesenchymal markers in these cultures are derived by EMT from beta cells. EMT has been documented during embryonic development and in pathological conditions, such as tumor progression and fibrosis. However, to our knowledge this is the first demonstration based on genetic lineage-tracing of EMT in cultured adult primary human cells.

EMT in cultured islet beta cells is likely a consequence of cell dissociation from the normal epithelial structure, resulting in altered cell-cell and cell-matrix contacts. In intact islets cultured on adherent surfaces this dissociation occurs gradually, and activation of mesenchymal marker expression is observed within several weeks in cells migrating out of the islet cell cluster [16]. In our islet cell expansion protocol, which involves trypsinization of isolated islets into a single-cell suspension prior to plating, this is a rapid process, leading to appearance of mesenchymal markers in the vast majority of the cells within several days [8], [20]. The loss of normal cell-cell and cell-matrix contacts likely leads to intracellular signaling events resulting in profound changes in gene expression, including loss of expression of differentiated beta-cell markers and activation of mesenchymal and MSC markers. It is not known whether EMT is required for induction of cell replication. The switch from E-cadherin to N-cadherin, which is a hallmark of EMT, has been shown to be associated with activation of the WNT pathway and regulation of cell proliferation and differentiation in multiple systems [23]–[25]. We have shown that the NOTCH pathway, which is also turned on during EMT [26], is activated in cultured human beta cells, and its activation is required for induction of cell proliferation [27]. However, it is possible that the state of responsiveness to replication cues from culture medium components is induced in these cells in part independently of EMT, by other changes in gene expression triggered by cell culture.

Despite studies on cell cycle regulation in beta cells (see refs. 28 and 29 for reviews), our understanding of the relations between beta-cell replication and expression of differentiated functions remains limited. It is unknown whether differentiated beta cells in human pancreas *in vivo* must undergo temporary dedifferentiation/EMT before entering cell cycle. Nevertheless, it appears that induction of significant beta-cell replication *in vitro* requires cell delamination out of the normal epithelial structure, a process that results in EMT, as revealed here by cell lineage tracing. In the context of *ex vivo* expansion of human islet beta cells for eventual use in cell therapy of diabetes, this dedifferentiation may be an unavoidable price to pay for increasing cell mass. We hypothesize that the dedifferentiated beta cells may retain epigenetic modifications in loci important for the beta-cell phenotype, making these loci relatively accessible to transcription, compared with cells from other tissues. However, transcription may be prevented by loss of expression of transcription activators, and upregulation of transcription repressors, resulting from cell delamination and replication. Once the normal repertoire of transcription activation and repression factors is restored by shifting the culture to appropriate differentiation conditions, the permissive chromatin structure may facilitate re-establishment of the beta-cell phenotype. This hypothesis is supported by our findings of partial restoration of beta-cell gene expression *in vitro*, obtained using relatively simple manipulations [20], [27], and is currently a central focus of research in our laboratory.

In addition to mesenchymal markers, culturing of human islet cells induced expression of several markers which are characteristic of the MSC phenotype (CD73, CD90, and CD105) in the majority of the cells. However, our results show that on average only 5.5% of the expanded islet cells at different passages can be viewed as *bona fide* MSC, as judged by their ability to differentiate into adipocytes or osteocytes. Moreover, very few of these cells (1.4%) were derived from beta cells. These findings were reproducible with 3 different commercial differentiation kits. These results differ from previous work, which reported considerable differentiation into mesodermal cell types of MSC-like cells derived from islet cells expanded *in vitro* [16], [18]. However, these results are difficult to compare to ours, since these groups did not provide quantitative data. In addition, it is possible that the culture method employed in these studies, which plated intact islets, as opposed to the initial islet dissociation employed in our protocol, results in preferential expansion of a different cell population. This possibility is supported by differences observed between cells expanded by the two protocols in doubling time (about 7 days in our protocol, compared with 2.5 days in the Gershengorn protocol) and overall expansion rate (<10^5^-fold in our protocol, compared with 10^12^-fold in the Gershengorn protocol). Our findings indicate that EMT does not induce multipotency in the dedifferentiated beta cells, suggesting that they retain a restricted differentiation potential and may be amenable to redifferentiation into functional beta cells.

## References

[1] Dor Y, Brown J, Martinez OI, Melton DA (2004). Adult pancreatic beta-cells are formed by self-duplication rather than stem-cell differentiation.. Nature.

[2] Teta M, Rankin MM, Long SY, Stein GM, Kushner JA (2007). Growth and regeneration of adult beta cells does not involve specialized progenitors.. Dev Cell.

[3] Nir T, Melton DA, Dor Y (2007). Recovery from diabetes in mice by beta cell regeneration.. J Clin Invest.

[4] Cano DA, Rulifson IC, Heiser PW, Swigart LB, Pelengaris S (2008). Regulated beta-cell regeneration in the adult mouse pancreas.. Diabetes.

[5] Hayek A, Beattie GM, Cirulli V, Lopez AD, Ricordi C (1995). Growth factor/matrix-induced proliferation of human adult beta-cells.. Diabetes.

[6] Beattie GM, Cirulli V, Lopez AD, Hayek A (1997). Ex vivo expansion of human pancreatic endocrine cells.. J Clin Endocrinol Metab.

[7] Beattie GM, Itkin-Ansari P, Cirulli V, Leibowitz G, Lopez AD (1999). Sustained proliferation of PDX-1+ cells derived from human islets.. Diabetes.

[8] Russ HA, Bar Y, Ravassard P, Efrat S (2008). In vitro proliferation of cells derived from adult human beta cells revealed by cell-lineage tracing.. Diabetes.

[9] Weinberg N, Ouziel-Yahalom L, Knoller S, Efrat S, Dor Y (2007). Lineage tracing evidence for in vitro dedifferentiation but rare proliferation of mouse pancreatic beta-cells.. Diabetes.

[10] Chase LG, Ulloa-Montoya F, Kidder BL, Verfaillie CM (2007). Islet-derived fibroblast-like cells are not derived via epithelial-mesenchymal transition from Pdx-1 or insulin-positive cells.. Diabetes.

[11] Atouf F, Park CH, Pechhold K, Ta M, Choi Y (2007). No evidence for mouse pancreatic beta-cell epithelial-mesenchymal transition in vitro.. Diabetes.

[12] Morton RA, Geras-Raaka E, Wilson LM, Raaka BM, Gershengorn MC (2007). Endocrine precursor cells from mouse islets are not generated by epithelial-to-mesenchymal transition of mature beta cells.. Mol Cell Endocrinol.

[13] Ulianich L, Garbi C, Treglia AS, Punzi D, Miele C (2008). ER stress is associated with dedifferentiation and an epithelial-to-mesenchymal transition-like phenotype in PC Cl3 thyroid cells.. J Cell Sci.

[14] Voulgari A, Pintzas A (2009). Epithelial-mesenchymal transition in cancer metastasis: Mechanisms, markers and strategies to overcome drug resistance in the clinic.. Biochim Biophys Acta.

[15] Mani SA, Guo W, Liao MJ, Eaton EN, Ayyanan A (2008). The epithelial-mesenchymal transition generates cells with properties of stem cells.. Cell.

[16] Gershengorn MC, Hardikar AA, Wei C, Geras-Raaka E, Marcus-Samuels B (2004). Epithelial-to-mesenchymal transition generates proliferative human islet precursor cells.. Science.

[17] Davani B, Ikonomou L, Raaka BM, Geras-Raaka E, Morton RA (2007). Human islet-derived precursor cells are mesenchymal stromal cells that differentiate and mature to hormone-expressing cells in vivo.. Stem Cells.

[18] Gallo R, Gambelli F, Gava B, Sasdelli F, Tellone V (2007). Generation and expansion of multipotent mesenchymal progenitor cells from cultured human pancreatic islets.. Cell Death Differ.

[19] Seeberger KL, Dufour JM, Shapiro AM, Lakey JR, Rajotte RV (2006). Expansion of mesenchymal stem cells from human pancreatic ductal epithelium.. Lab Invest.

[20] Ouziel-Yahalom L, Zalzman M, Anker-Kitai L, Knoller S, Bar Y (2006). Expansion and redifferentiation of adult human pancreatic islet cells.. Biochem Biophys Res Commun.

[21] Karnieli O, Izhar-Prato Y, Bulvik S, Efrat S (2007). Generation of insulin-producing cells from human bone marrow mesenchymal stem cells by genetic manipulation.. Stem Cells.

[22] Cabrera O, Berman DM, Kenyon NS, Ricordi C, Berggren PO (2006). The unique cytoarchitecture of human pancreatic islets has implications for islet cell function.. Proc Natl Acad Sci USA.

[23] Shah GV, Muralidharan A, Gokulgandhi M, Soan K, Thomas S (2009). Cadherin switching and activation of beta-catenin signaling underlie proinvasive actions of calcitonin-calcitonin receptor axis in prostate cancer.. J Biol Chem.

[24] Lim YS, Lee HC, Lee HS (2007). Switch of cadherin expression from E- to N-type during the activation of rat hepatic stellate cells.. Histochem Cell Biol.

[25] Hazan RB, Qiao R, Keren R, Badano I, Suyama K (2004). Cadherin switch in tumor progression.. Ann N Y Acad Sci.

[26] Wang Z, Li Y, Kong D, Ahmad A, Azmi AS (2009). Acquisition of epithelial-mesenchymal transition phenotype of gemcitabine-resistant pancreatic cancer cells is linked with activation of the notch signaling pathway.. Cancer Res.

[27] Bar Y, Russ HA, Knoller S, Ouziel-Yahalom L, Efrat S (2008). HES1 is involved in adaptation of adult human beta cells to proliferation in vitro.. Diabetes.

[28] Cozar-Castellano I, Fiaschi-Taesch N, Bigatel TA, Takane KK, Garcia-Ocaña A (2006). Molecular control of cell cycle progression in the pancreatic beta-cell.. Endocr Rev.

[29] Heit JJ, Karnik SK, Kim SK (2006). Intrinsic regulators of pancreatic beta-cell proliferation.. Annu Rev Cell Dev Biol.

